# Distribution of waist-to-height ratio and cardiometabolic risk in children and adolescents: a population-based study

**DOI:** 10.1038/s41598-021-88951-9

**Published:** 2021-05-04

**Authors:** Hye Jin Lee, Young Suk Shim, Jong Seo Yoon, Hwal Rim Jeong, Min Jae Kang, Il Tae Hwang

**Affiliations:** 1 Department of Pediatrics, Hallym University Kangnam Sacred Heart Hospital, Seoul, Republic of Korea; 2 Department of Pediatrics, Ajou University School of Medicine, Ajou University Hospital, Suwon, Korea; 3 Department of Pediatrics, Kangdong Sacred Heart Hospital, Seoul, Republic of Korea; 4 Department of Pediatrics, Soonchunhyang University Cheonan Hospital, Soonchunhyang University College of Medicine, Cheonan, Korea; 5 Department of Pediatrics, Hallym University Sacred Heart Hospital, Anyang, Republic of Korea

**Keywords:** Cardiology, Endocrinology, Health care, Medical research, Risk factors

## Abstract

This study was performed to evaluate the waist-to-height ratio (WHtR) distribution and assess its relationship with cardiometabolic risk in children and adolescents. A total of 8091 subjects aged 10–18 years were included from a nationally representative survey. Participants were classified into three groups: (1) < 85th, (2) ≥ 85th and < 95th, and (3) ≥ 95th percentile of WHtR. The WHtR distribution varied with sex and age. Whereas WHtR decreased from age 10–15 years in boys and from age 10–12 years in girls, it slightly increased thereafter. Compared to the < 85th percentile group, the WHtR ≥ 85th and < 95th percentile group had an odds ratio (OR) of 1.2 for elevated blood pressure (BP), 1.89 for elevated triglycerides (TGs), 1.47 for reduced high-density lipoprotein cholesterol (HDL-C) and 4.82 for metabolic syndrome (MetS). The ≥ 95th percentile group had an OR of 1.4 for elevated BP, 2.54 for elevated glucose, 2.22 for elevated TGs, 1.74 for reduced HDL-C, and 9.45 for MetS compared to the < 85th percentile group. Our results suggest that sex- and age-specific WHtR percentiles can be used as a simple clinical measurement to estimate cardiometabolic risk.

## Introduction

Childhood obesity is increasing worldwide^1^. As the prevalence of childhood obesity increases, metabolic syndrome (MetS), which is a clustering of abdominal obesity-associated factors including elevated waist circumference (WC), elevated blood pressure (BP), elevated glucose, elevated triglycerides (TGs) or reduced high-density lipoprotein cholesterol (HDL-C), has also increased^2^. Obesity in children and adolescents commonly progresses to adult obesity and increases the likelihood of comorbidities associated with cardiometabolic risk, such as type 2 diabetes mellitus (T2DM), dyslipidemia, and hypertension^3,4^. This high level of progression makes screening a time-sensitive issue for children at high risk for current and future cardiometabolic problems, as the window for intervention might be short.

Obesity in children and adolescents is defined as a body mass index (BMI) above the 95th percentile for sex and age. While BMI is a simple measure calculated by dividing the body weight by the squared height, it does not fully reflect adiposity or body composition. Since central obesity is known to be an indicator of cardiometabolic risk, better anthropometric measures than BMI to screen for central obesity are needed. Among the anthropometric measures used to evaluate adiposity, the waist-to-height ratio (WHtR) can be easily measured in clinical settings and can act as an indicator of central adiposity. WHtR, which is WC (cm) divided by height (cm), has been proposed as a strong predictor of cardiometabolic risk. WHtR has been found to be superior to BMI or WC when predicting metabolic diseases such as hypertension, T2DM, dyslipidemia and MetS in adults and children^5–9^.

A WHtR above 0.5 is now recognized as a risk factor for cardiometabolic diseases in adults^8^. However, the use of a single WHtR threshold for cardiometabolic risk in children and adolescents has been debated^10–12^. Since height and WC vary according to sex and age in children and adolescents, percentiles or standard deviation scores (SDSs) rather than a single cut-off have been proposed to evaluate cardiometabolic risk. Similarly, sex- and age-specific BMI percentiles are used to define obesity in children and adolescents, while a single cut-off value of 25 kg/m^2^ is used for adults. While a previous study reported on the centile charts and secular trend of WHtR in Korean children and adolescents^13^, there have been no publications on sex- and age-specific LMS values to calculate SDSs or exact centiles of WHtR in Korean children despite such data already having been accumulated in other countries^12,14^.

Therefore, we aimed to evaluate the distribution of WHtR and provide a sex- and age-specific reference for WHtR using the LMS method in Korean children and adolescents using a nationally representative survey, the Korea National Health and Nutrition Examination Survey (KNHANES). We also compared the adjusted mean values of cardiometabolic risk factors and adjusted odds ratios (ORs) for MetS and its components between groups according to WHtR percentiles to determine whether WHtR percentiles can be used to predict cardiometabolic risk.

## Results

### Clinical characteristics of the study population according to sex

The clinical characteristics of the study population (*n* = 8091) are shown in Table 1. The mean age of the total population was 14.33 ± 2.51 years, and the mean BMI SDS was -0.04 ± 1.24. The SBP ≥ 90th percentile group accounted for 3.5% of the total population (*n* = 288), and the DBP ≥ 90th percentile group accounted for 23.2% (*n* = 2361). Compared to girls, boys had higher mean values for height SDS (*P* = 0.004), WC SDS (*P* = 0.029), WHtR (*P* < 0.001), systolic blood pressure (SBP) (*P* < 0.001), diastolic blood pressure (DBP) (*P* < 0.001), and glucose levels (*P* < 0.001), whereas they had lower mean values for WC SDS (*P* = 0.004), and total cholesterol (T-C) (*P* < 0.001), HDL-C (*P* < 0.001), TG (*P* = 0.001) and low-density lipoprotein cholesterol (LDL-C) (*P* < 0.001) levels. Boys were more likely to drink alcohol, smoke, and be physically active (all *P* < 0.001).

**Table 1:** Clinical characteristics of the study population (*n* = 8091).

	Total	Boys	Girls	*P*
	*n* = 8091	*n* = 4306	*n* = 3785	
Age (years)	14.33 ± 2.51	14.30 ± 2.51	14.36 ± 2.51	0.257
Height SDS	0.22 ± 1.05	0.25 ± 1.05	0.19 ± 1.05	0.004
Weight SDS	0.08 ± 1.18	0.10 ± 1.22	0.05 ± 1.14	0.081
WC SDS	− 0.22 ± 1.1	− 0.24 ± 1.13	− 0.19 ± 1.09	0.029
BMI SDS	− 0.04 ± 1.24	− 0.04 ± 1.29	− 0.05 ± 1.19	0.643
WHtR	0.43 ± 0.05	0.44 ± 0.06	0.43 ± 0.05	< 0.001
SBP (mmHg)	106.65 ± 10.27	108.80 ± 10.60	104.21 ± 9.30	< 0.001
**SBP percentile**				
< 50th percentile (%)	5241 (64.8%)	2881 (66.9%)	2360 (62.4%)	< 0.001
50th–90th percentile (%)	2562 (31.7%)	1303 (30.3%)	1259 (33.3%)	
90th–95th percentile (%)	164 (2.0%)	65 (1.5%)	99 (2.6%)	
≥ 95th percentile (%)	124 (1.5%)	57 (1.3%)	67 (1.7%)	
DBP (mmHg)	66.00 ± 9.04	66.39 ± 9.64	65.56 ± 8.28	< 0.001
**DBP percentile**				
< 50th percentile (%)	2128 (26.3%)	1110 (25.8%)	1018 (26.9%)	< 0.001
50th–90th percentile (%)	3602 (44.5%)	1858 (43.2%)	1744 (46.1%)	
90th–95th percentile (%)	922 (11.4%)	497 (11.5%)	425 (11.2%)	
≥ 95th percentile (%)	1439 (11.8%)	841 (19.5%)	598 (15.8%)	
Glucose (mg/dL)	90.18 ± 8.52	90.83 ± 7.98	89.43 ± 9.04	< 0.001
T-C (mg/dL)	159.55 ± 27.01	155.80 ± 27.07	163.82 ± 26.30	< 0.001
HDL-C (mg/dL)	50.97 ± 9.95	49.86 ± 9.91	52.22 ± 9.86	< 0.001
TGs (mg/dL)	84.70 ± 45.92	83.04 ± 47.31	86.57 ± 44.22	0.001
LDL-C (mg/dL)	91.68 ± 23.21	89.39 ± 23.25	94.27 ± 22.89	< 0.001
Drinkers	2037 (25.18%)	1172 (27.2%)	865 (22.9%)	< 0.001
Smokers	930 (11.49%)	681 (15.8%)	249 (6.6%)	< 0.001
Physical activity	4604 (56.90%)	2515 (58.4%)	2089 (55.2%)	0.004
Rural residence	1311 (16.20%)	699 (16.2%)	612 (16.2%)	0.962
Low household income	885 (10.94%)	467 (10.9%)	418 (11.0%)	0.803
T2DM	4 (0.05%)	2 (0.1%)	2 (0.1%)	> 0.999
Hypertension	0 (0%)	0 (0%)	0 (0%)	> 0.999
Dyslipidemia	0 (0%)	0 (0%)	0 (0%)	> 0.999

The results are expressed as the mean ± standard deviation (SD) or n (%).

Drinkers consumed at least two alcoholic beverages/month during the previous year; smokers smoked more than five packs of cigarettes throughout their lives; physically active individuals met at least one of the following criteria: (1) intense physical activity for 20 min at least three days/week, (2) moderate physical activity for 30 min at least five days/week, or (3) walking for 30 min at least five days/week.

*SDS* standard deviation score, *WHtR* waist circumference-to-height ratio, *BMI* body mass index, *SBP* systolic blood pressure, *DBP* diastolic blood pressure, *T-C* total cholesterol, *HDL-C* high-density lipoprotein cholesterol, *TGs* triglycerides, *LDL-C* low-density lipoprotein cholesterol, *T2DM* type 2 diabetes mellitus.

### Sex- and age-specific distribution of WHtR

**Figure 1:**
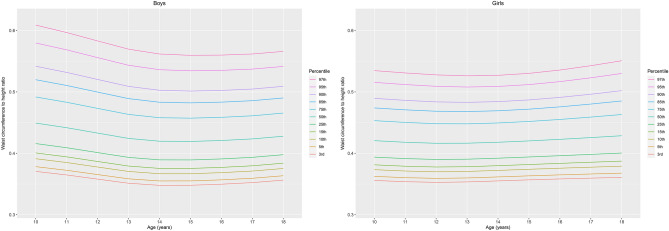
Percentile curves of the age- and sex-specific waist-to-height ratio of children and adolescents aged 10–18 years.

LMS and percentile values at the 3rd, 10th, 15th, 25th, 50th, 75th, 85th, 90th, and 97th percentiles according to sex and age are shown in Table 2 and Fig. 1. WHtR varied considerably according to sex and age. Both boys and girls had U-shaped percentile curves according to age, and these curves were skewed to the left. WHtR decreased from age 10–15 years in boys and from age 10 to approximately 12 years in girls and slightly increased thereafter. Variations in WHtR according to age were higher in the upper percentile groups (≥ 85th percentiles) than in the lower percentile groups (3rd–50th percentiles). Boys had higher WHtRs than girls in the younger age groups (10–14 years) and lower WHtR in the lower percentile groups after 14 years. Boys had higher coefficients of variation in all age groups.

**Table 2:** Distribution of waist circumference-to-height ratio (WHtR) in Korean children and adolescents according to sex (*n* = 8091).

Age	*N*	L	M	S	3rd	5th	10th	15th	25th	50th	75th	85th	90th	95th	97th
**Boys**															
10	476	− 1.869	0.449	0.123	0.371	0.378	0.391	0.401	0.416	0.449	0.492	0.520	0.542	0.580	0.609
11	516	− 1.869	0.442	0.122	0.365	0.372	0.385	0.394	0.409	0.442	0.483	0.510	0.532	0.569	0.597
12	524	− 1.869	0.433	0.121	0.358	0.365	0.378	0.387	0.401	0.433	0.473	0.500	0.520	0.556	0.583
13	543	− 1.869	0.424	0.120	0.351	0.358	0.370	0.379	0.393	0.424	0.463	0.489	0.509	0.543	0.570
14	524	− 1.869	0.420	0.119	0.348	0.355	0.367	0.375	0.389	0.420	0.458	0.483	0.503	0.536	0.562
15	480	− 1.869	0.419	0.119	0.348	0.355	0.367	0.375	0.389	0.419	0.457	0.482	0.501	0.534	0.560
16	421	− 1.869	0.421	0.118	0.350	0.357	0.369	0.377	0.391	0.421	0.459	0.483	0.502	0.535	0.560
17	427	− 1.869	0.423	0.117	0.352	0.359	0.371	0.380	0.393	0.423	0.461	0.486	0.505	0.537	0.562
18	395	− 1.869	0.428	0.116	0.356	0.363	0.375	0.384	0.398	0.428	0.466	0.490	0.509	0.541	0.566
**Girls**															
10	415	− 1.798	0.421	0.104	0.356	0.362	0.373	0.381	0.394	0.421	0.453	0.474	0.490	0.516	0.535
11	444	− 1.798	0.418	0.103	0.354	0.360	0.371	0.379	0.391	0.418	0.450	0.471	0.486	0.512	0.531
12	431	− 1.798	0.416	0.103	0.353	0.359	0.370	0.378	0.390	0.416	0.448	0.469	0.484	0.509	0.528
13	464	− 1.798	0.416	0.102	0.353	0.360	0.371	0.378	0.390	0.416	0.448	0.468	0.483	0.508	0.526
14	469	− 1.798	0.418	0.101	0.355	0.362	0.372	0.380	0.392	0.418	0.449	0.469	0.484	0.509	0.527
15	399	− 1.798	0.420	0.101	0.357	0.363	0.374	0.382	0.394	0.420	0.452	0.472	0.487	0.512	0.530
16	414	− 1.798	0.423	0.102	0.358	0.365	0.376	0.384	0.396	0.423	0.455	0.476	0.491	0.517	0.536
17	421	− 1.798	0.426	0.105	0.360	0.366	0.377	0.385	0.398	0.426	0.459	0.480	0.496	0.523	0.543
18	328	− 1.798	0.429	0.107	0.361	0.368	0.379	0.387	0.400	0.429	0.463	0.485	0.502	0.530	0.551

### Comparison of adjusted mean values for cardiometabolic risk factors according to the WHtR percentiles

Adjusted mean values of cardiometabolic risk factors according to the WHtR groups after adjustment for possible confounders are shown in Table 3. For all participants, the group with WHtR ≥ 85th and < 95th percentile had higher adjusted means T-C (*P* < 0.001), TGs (*P* < 0.001), and LDL-C (*P* = 0.002); the group with WHtR ≥ 85th and < 95th percentile had lower HDL-C levels (*P* < 0.001) than the group with WHtR < 85th percentile. Compared to the WHtR < 85th percentile group, the WHtR ≥ 95th percentile group had higher adjusted means for T-C (*P* < 0.001), TGs (*P* < 0.001), and LDL-C (*P* < 0.001), whereas the WHtR ≥ 95th percentile group exhibited lower adjusted means for HDL-C (*P* < 0.001). In addition, the WHtR ≥ 95th percentile group had higher adjusted means for TGs (*P* = 0.021) than the group with WHtR ≥ 85th and < 95th percentile.

**Table 3:** The adjusted means of cardiometabolic risk factors according to waist circumference-to-height ratio (WHtR) in Korean children and adolescents (*n* = 8091).

	WC-to-height ratio		
	< 85th percentile	≥ 85th and < 95th percentile	≥ 95th percentile
**All participants**	*n* = 6746	*n* = 939	*n* = 406
WC SDS	− 0.28 ± 0.01	0.06 ± 0.02^a^	0.29 ± 0.03^b,c^
SBP (mmHg)	106.54 ± 0.12	107.16 ± 0.35	107.32 ± 0.54
DBP (mmHg)	65.89 ± 0.11	66.41 ± 0.32	66.82 ± 0.50
Glucose (mg/dL)	90.14 ± 0.11	90.10 ± 0.31	91.03 ± 0.48
T-C (mg/dL)	158.51 ± 0.34	163.77 ± 0.98^a^	167.09 ± 1.52^b^
HDL-C (mg/dL)	51.35 ± 0.12	49.22 ± 0.36^a^	48.47 ± 0.55^b^
TGs (mg/dL)	81.29 ± 0.57	99.59 ± 1.64^a^	106.81 ± 2.57^b,c^
LDL-C (mg/dL)	90.89 ± 0.29	94.70 ± 0.85^a^	97.46 ± 1.32^b^
**Boys**	*n* = 3573	*n* = 534	*n* = 199
WC SDS	− 0.28 ± 0.01	− 0.08 ± 0.02^a^	− 0.08 ± 0.04^b^
SBP (mmHg)	108.82 ± 0.17	109.13 ± 0.47	109.35 ± 0.79
DBP (mmHg)	66.26 ± 0.16	67.47 ± 0.45^a^	67.65 ± 0.75
Glucose (mg/dL)	90.99 ± 0.14	90.11 ± 0.38	90.19 ± 0.64
T-C (mg/dL)	154.29 ± 0.47	162.27 ± 1.30^a^	168.58 ± 2.18^b,c^
HDL-C (mg/dL)	50.19 ± 0.17	48.32 ± 0.47^a^	48.30 ± 0.79
TGs (mg/dL)	79.42 ± 0.81	99.40 ± 2.26^a^	106.98 ± 3.79^b^
LDL-C (mg/dL)	88.21 ± 0.40	94.27 ± 1.13^a^	99.18 ± 1.88^b,c^
**Girls**	*n* = 3173	*n* = 405	*n* = 207
WC SDS	− 0.29 ± 0.19	0.20 ± 0.03^a^	0.61 ± 0.04^b,c^
SBP (mmHg)	104.08 ± 0.17	104.56 ± 0.50	104.27 ± 0.72
DBP (mmHg)	65.62 ± 0.15	65.03 ± 0.45	65.61 ± 0.64
Glucose (mg/dL)	89.20 ± 0.17	89.95 ± 0.49	91.69 ± 0.71^b^
T-C (mg/dL)	163.14 ± 0.50	165.82 ± 1.46	168.21 ± 2.11
HDL-C (mg/dL)	52.57 ± 0.18	50.55 ± 0.53^a^	49.73 ± 0.77^b^
TGs (mg/dL)	83.86 ± 0.82	98.68 ± 2.39^a^	105.22 ± 3.45^b^
LDL-C (mg/dL)	93.77 ± 0.44	95.46 ± 1.27	97.48 ± 1.84

The results are expressed as the mean ± standard error (SE).

The adjusted means of cardiometabolic risk factors were estimated using analysis of covariance (ANCOVA) with Bonferroni’s post hoc test after adjustment for age, sex, body mass index (BMI) standard deviation score (SDS), alcohol consumption, smoking, physical activity, residence, household income, and diagnosis of hypertension, diabetes mellitus and dyslipidemia.

*WC* waist circumference, *SDS* standard deviation score, *BMI* body mass index, *SBP* systolic blood pressure, *DBP* diastolic blood pressure, *T-C* total cholesterol, *HDL-C* high-density lipoprotein cholesterol, *TGs* triglycerides, *LDL-C* low-density lipoprotein cholesterol.

^a^The difference was estimated between the < 85th percentile group and the ≥ 85th and < 95th percentile group using analysis of covariance with Bonferroni’s post hoc test.

^b^The difference was estimated between the < 85th percentile group and the ≥ 95th percentile group using analysis of covariance with Bonferroni’s post hoc test.

^c^The difference was estimated between the ≥ 85th and < 95th percentile group and the ≥ 95th percentile group using analysis of covariance with Bonferroni’s post hoc test.

In the subgroup analyses, boys in the WHtR ≥ 85th and < 95th percentile group had higher DBP (*P* = 0.046), T-C (*P* < 0.001), TGs (*P* < 0.001), and LDL-C (*P* < 0.001) and lower HDL-C (*P* = 0.001) levels than the WHtR < 85th percentile group. Boys in the WHtR ≥ 95th percentile group had higher means for T-C (*P* < 0.001), TGs (*P* < 0.001), and LDL-C (*P* < 0.001) than the WHtR < 85th percentile group. Boys in the WHtR ≥ 95th percentile group had higher means for T-C (*P* = 0.013) and LDL-C (*P* = 0.030) than the WHtR ≥ 85th and < 95th percentile group. Girls in the WHtR ≥ 85th and < 95th percentile group had higher adjusted means for TGs (*P* < 0.001) but lower adjusted means for HDL-C levels (*P* = 0.002) than the WHtR < 85th percentile group. Girls in the WHtR ≥ 95th percentile group had higher means for glucose (*P* = 0.003) and TGs (*P* < 0.001) but lower means for HDL-C levels (*P* = 0.002) than the WHtR < 85th percentile group.

### Adjusted OR for MetS and its components according to the WHtR percentile groups

Adjusted ORs for MetS and its components according to the WHtR percentiles are shown in Table 4. Compared with those in the WHtR < 85th percentile group, the subjects in the WHtR ≥ 85th and < 95th percentile group exhibited increased ORs (95% confidence intervals, CIs) of 24.40 (15.25–39.03) for elevated WC, 1.20 (1.01–1.43) for elevated BP, 1.89 (1.58–2.27) for elevated TGs, 1.47 (1.18–1.83) for reduced HDL-C, and 4.82 (3.35–6.94) for MetS. Subjects in the WHtR ≥ 95th percentile group exhibited increased ORs of 214.43 (116.37–396.22) for elevated WC, 1.40 (1.09–1.81) for elevated BP, 2.54 (1.01–6.42) for elevated glucose, 2.22 (1.70–2.90) for elevated TGs, 1.74 (1.28–2.38) for reduced HDL-C, and 9.45 (5.84–15.27) for MetS compared with the WHtR < 85th percentile group.

**Table 4:** The adjusted odds ratio of metabolic syndrome (MetS) and its components according to waist circumference-to-height ratio (WHtR) in Korean children and adolescents (*n* = 8091).

	WC to height ratio		
	< 85th percentile	≥ 85th and < 95th percentile	≥ 95th percentile
**All participants**	*n* = 6746	*n* = 939	*n* = 406
Elevated WC	Reference	24.40 (15.25–39.03)	214.43 (116.37–396.22)
Elevated BP	Reference	1.20 (1.01–1.43)	1.40 (1.09–1.81)
Elevated glucose	Reference	0.80 (0.35–1.83)	2.54 (1.01–6.42)
Elevated TGs	Reference	1.89 (1.58–2.27)	2.22 (1.70–2.90)
Reduced HDL-C	Reference	1.47 (1.18–1.83)	1.74 (1.28–2.38)
MetS	Reference	4.82 (3.35–6.94)	9.45 (5.84–15.27)
**Boys**	*n* = 3573	*n* = 534	*n* = 199
Elevated WC	Reference	27.12 (11.37–64.68)	217.14 (73.12–644.85)
Elevated BP	Reference	1.42 (1.12–1.79)	1.52 (1.05–2.20)
Elevated glucose	Reference	0.46 (0.14–1.53)	1.66 (0.44–6.20)
Elevated TGs	Reference	2.02 (1.57–2.60)	2.17 (1.47–3.21)
Reduced HDL-C	Reference	1.52 (1.15–2.01)	1.48 (0.97–2.27)
MetS	Reference	3.92 (2.46–6.24)	5.86 (3.06–11.23)
**Girls**	*n* = 3173	*n* = 405	*n* = 207
Elevated WC	Reference	32.13 (17.90–57.68)	339.89 (154.49–747.78)
Elevated BP	Reference	0.92 (0.71–1.20)	1.16 (0.81–1.65)
Elevated glucose	Reference	1.64 (0.50–5.42)	4.73 (1.22–18.36)
Elevated TGs	Reference	1.65 (1.26–2.16)	2.06 (1.42–2.97)
Reduced HDL-C	Reference	1.28 (0.90–1.82)	1.79 (1.13–2.83)
MetS	Reference	6.11 (3.38–11.04)	15.37 (7.46–31.67)

The adjusted odds ratio and 95% confidence interval were estimated using multiple logistic regression analysis after adjustment for age, sex, body mass index (BMI) standard deviation score (SDS), alcohol consumption, smoking, physical activity, residence, household income, and diagnosis of hypertension, diabetes mellitus and dyslipidemia.

*WC* waist circumference, *BP* blood pressure, *TGs* triglycerides, *HDL-C* high-density lipoprotein cholesterol, *MetS* metabolic syndrome.

When stratified by sex, boys in the WHtR ≥ 85th and < 95th percentile group exhibited increased ORs (95% CI) of 27.12 (11.37–64.68) for elevated WC, 1.42 (1.12–1.79) for elevated BP, 2.02 (1.57–2.60) for elevated TGs, 1.52 (1.15–2.01) for reduced HDL-C, and 3.92 (2.46–6.24) for MetS compared with boys in the WHtR < 85th percentile group. Boys in the WHtR ≥ 95th percentile group showed increased ORs (95% CI) of 217.14 (73.12–644.85) for elevated WC, 1.52 (1.05–2.20) for elevated BP, 2.17 (1.47–3.21) for elevated TGs, and 5.86 (3.06–11.23) for MetS compared with boys in the WHtR < 85th percentile group. For girls, the WHtR ≥ 85th and < 95th percentile group exhibited increased ORs (95% CI) of 32.13 (17.90–57.68) for elevated WC, 1.65 (1.26–2.16) for elevated TGs, and 6.11 (3.38–11.04) for MetS compared with girls in the WHtR < 85th percentile group. Girls in the WHtR ≥ 95th percentile group showed increased ORs (95% CI) of 339.89 (154.49–747.78) for elevated WC, 4.73 (1.22–18.36) for elevated glucose, 2.06 (1.42–2.97) for elevated TGs, 1.79 (1.13–2.83) for reduced HDL-C, and 15.37 (7.46–31.67) for MetS compared with girls in the WHtR < 85th percentile group.

## Discussion

This nationally representative population-based study demonstrated the sex- and age-specific distribution of WHtR. In the present study, we found U-shaped percentile curves that mostly decreased throughout childhood with variation according to sex and age. Analyses of covariance (ANCOVA) showed that sex- and age-specific WHtRs were positively correlated with WC SDS, T-C, TGs, and LDL-C, whereas they were inversely correlated with HDL-C. Our multiple logistic regression analysis found that boys and girls with WHtR ≥ 85th and < 95th percentile exhibited increased ORs for elevated WC, elevated BP, elevated TGs, reduced HDL-C and MetS, whereas subjects with WHtR ≥ 95th percentile had higher ORs for elevated WC, elevated BP, elevated glucose, elevated TGs, reduced HDL-C and MetS than subjects with WHtR < 85th percentile. Compared to the WHtR < 85th percentile group, the WHtR ≥ 85th and < 95th percentile group showed moderate increases, and the WHtR ≥ 95th percentile group showed higher increases in the mean levels of cardiometabolic risk factors and adjusted ORs for MetS and its components.

Though WHtR is a simple anthropometric measure of central obesity used in clinical settings, unlike in adults, the distribution of WHtR may vary in children and adolescents since they grow and their body proportions change during puberty^15^. In previous studies with Norwegian^11^, multiethnic U.S.^12^, Japanese^14^, Colombian^16^, and Yemeni^17^ children and adolescents, WHtR showed a U-shaped pattern according to age. Hong Kong Chinese children showed an L-shaped pattern for WHtR according to age, which decreased from age 6 to 14 years and was nearly constant thereafter^18^. Other studies in Greece^19^ and Pakistan^20^ showed continuously decreasing patterns of WHtR according to age. On the other hand, studies of Thai children^20^ and German adolescents^21^ showed no significant difference in WHtR according to age or sex. Our results show a U-shaped pattern for WHtR according to age, with a nadir presenting at a later age in boys in a group of Korean children and adolescents, as was observed in most of the previous studies. WHtR decreased to reach a minimum at age 15 in boys and 12 in girls and slightly increased thereafter. The later WHtR nadir may be associated with the later onset of puberty and growth spurts in boys than in girls. The age of the lowest WHtR was similar to the age of maximum growth velocity in Korean children and adolescents^22^. While most previous studies showed that boys have higher WHtRs in early ages^11,16^ or in all age groups^14,18,19^, a study with children from the U.S. showed that girls had higher WHtR than boys in all age groups^12^. In our study, boys had higher WHtRs than girls from 10 to 14 years of age in all percentile groups, and girls had higher WHtRs in the low percentile groups (≤ 50th percentile) after 14 years of age.

The variations in WHtR according to sex and age observed in this study contradict the use of a universal fixed cut-off for WHtR. McCarthy and Ashwell proposed a single fixed cut-off for WHtR and stated that a WHtR above 0.5 would suggest an increased risk for adverse health outcomes in children as in adults^23^. Other studies have also evaluated different fixed cut-offs to predict obesity or cardiometabolic risk^7,21,24,25^. However, as WHtR shows different distributions according to sex, age, and ethnicity, a single fixed cut-off point may not be appropriate for children and adolescents. Growing evidence suggests the need for sex- and age-specific WHtR percentile references and cut-offs to better evaluate cardiovascular risk in children and adolescents. A higher sex- and age-specific WHtR SDS was related to an increased risk of dyslipidemia and hyperglycemia in U.S. children^12^. Another study with Iranian school children^26^ suggested different cut-off values for components of MetS according to age. The WHtRs of Korean children and adolescents in the present study showed relatively stable patterns in the lower percentile groups (3rd to 50th percentiles), whereas the higher percentile groups (over the 85th percentile) had more variation according to sex and age. The variations in WHtR according to sex and age, especially in the higher percentile groups that were associated with cardiometabolic risk in this study, suggest that different cut-offs need to be established according to sex and age in children and adolescents.

Central obesity and cardiometabolic risks have been closely associated with each other. However, few studies have evaluated cardiometabolic risk according to WHtR percentiles. The cardiometabolic risks were evaluated according to the WHtR percentiles in children and adolescents. The age- and sex-specific 85th and 95th percentiles were adopted to assess the cardiometabolic risks, as is done for BMI percentile when defining childhood overweight (85th percentile ≤ BMI < 95th percentile) and obesity (BMI ≥ 95th percentile). In a previous study, the optimal cut-offs for WHtR SDS were the 85th and 95th percentiles for predicting central overweight and obesity, respectively, in Norwegian children and adolescents, although cardiometabolic risk was not assessed^11^. On the other hand, there was a study evaluating the relationship between WHtR percentile and cardiometabolic risks. A U.S. study evaluated cardiometabolic risk according to sex- and age-specific WHtR percentiles in children^12^. The ORs for elevated T-C, elevated LDL-C, decreased HDL-C, elevated TGs, and elevated glycated hemoglobin increased in association with a unit increase in WHtR SDS, which was considered a continuous variable; BP was not evaluated, and no cut-offs were provided for the WHtR percentiles when evaluating increased risk for adverse cardiometabolic factors in the previous study^12^. In our study, the risk for MetS increased in higher WHtR groups when the 85th and 95th percentiles were used as cut-offs. For each component of MetS, the risks for elevated WC, BP, and TGs and reduced HDL-C were higher in the higher WHtR percentile groups, whereas elevated glucose had a higher OR in only the ≥ 95th percentile group for the total population. To our knowledge, this is the first study to evaluate cardiometabolic risk and MetS components based on the National Cholesterol Education Program Adult Treatment Panel (NCEP ATP) III criteria according to central obesity groups defined according to sex- and age-specific WHtR percentiles in children and adolescents.

There are limitations to this study. First, the cross-sectional nature of this study prevented the assessment of causality. Second, although WC is a simple measurement, an additional measurement is needed to evaluate WHtR, and using a centile chart might be inconvenient in clinical settings. Third, we did not include children under 10 years of age due to a lack of biochemical measurements. As younger children show WHtR that varies drastically according to age^11,18,27^, further studies are needed in this age group. Finally, our study did not include data on the pubertal stage of each individual, which may influence both WHtR and metabolic outcomes as a confounder. Nevertheless, the difference in the age distribution of WHtR according to sex seems to reflect the age of puberty and growth spurt in Korean children. Since we evaluated the WHtR according to sex and age, the effect of puberty may be corrected to some extent.

In conclusion, the present study demonstrated sex- and age-specific LMS tables and percentile curves of WHtR in Korean children and adolescents using data from a nationally representative survey. The distribution of WHtR significantly varied according to sex and age based on sex- and age-specific percentiles. Based on these distributions, the relationship between groups according to WHtR percentiles and cardiometabolic risk was evaluated. The children and adolescents in the 85th-95th percentile WHtR were at high risk for elevated WC, elevated BP, elevated TGs, reduced HDL-C and MetS. Moreover, boys and girls in the ≥ 95th percentile of WHtR exhibited higher risks for elevated WC, elevated BP, elevated glucose, elevated TGs, reduced HDL-C, and MetS. Our results suggest that sex- and age-specific WHtR percentiles can appropriately estimate cardiometabolic risk and can be used in clinical settings in the pediatric population as a considerably simple measurement.

## Methods

### Subjects

**Figure 2:**
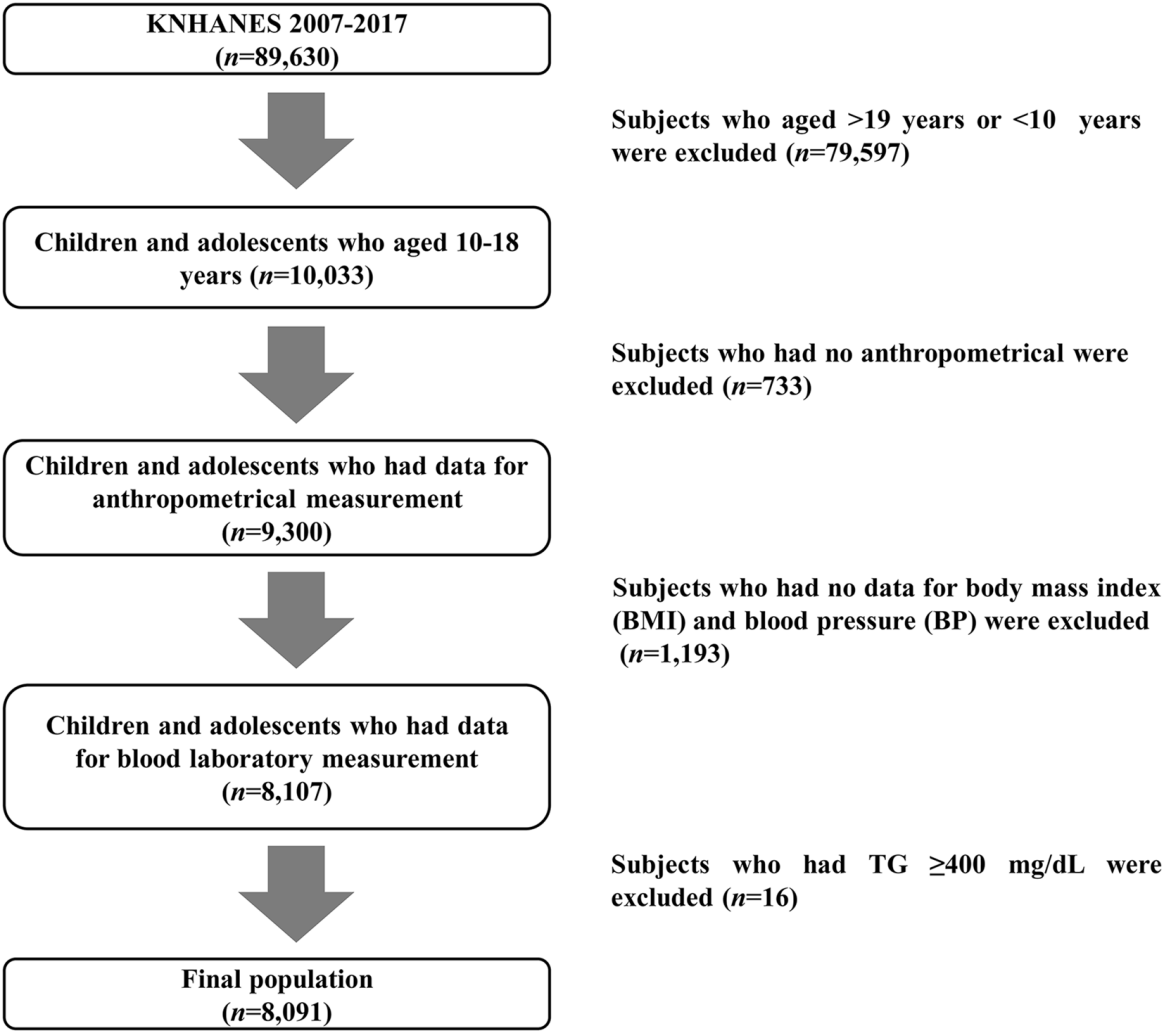
Flow chart of the study population.

The KNHANES data from 2007 to 2017 were analyzed in this study. The KNHANES is a cross-sectional, nationally representative survey that is regularly conducted by the Division of Chronic Disease Surveillance, Korean Centers for Disease Control and Prevention (KCDC) and is composed of a health questionnaire, health examination, and nutritional assessment; KNHANES uses a stratified and multistage probability sampling design to select household units for inclusion. Details of the KNHANES have been described previously^28^. Among the 89,630 subjects of KNHANES 2001–2017, children and adolescents aged 10–18 years were included in the initial analysis (*n* = 10,033). Subjects with missing anthropometrical measurements (*n* = 733) and missing blood laboratory results (*n* = 1193) were excluded. Participants with TGs ≥ 400 mg/dL (*n* = 16) were also excluded since the LDL-C levels were determined with Friedewald’s equation^29^. Finally, a total of 8091 children and adolescents were analyzed in this study (Fig. 2). The database is available to the public at the KNHANES website (http://knhanes.cdc.go.kr). All KNHANES subjects gave informed consent at the time of data collection and the methods used in the KNHANES were performed in accordance with relevant guidelines and regulations. This study was approved by the institutional review board (IRB) of Hallym University Kangnam Sacred Heart Hospital, and the requirement for participant consent was waived because public data was used that did not have identifying personal information (IRB No. 2021-03-007).

### Measurements

Anthropometric assessments were performed by a trained expert using standard methods. Height and body weight were assessed to the nearest 0.1 cm using a Seca 225 (Seca, Hamburg, Germany) and 0.1 kg using a GL-6000-20 (G-tech, Seoul, Korea), respectively. BMI (kg/m^2^) was calculated as weight (kg) divided by the square of height (m^2^). WC was measured at the midline between the lower rib margin and iliac crest to the nearest 0.1 cm. SDSs were used for height, weight, BMI, and WC due to the variable distribution of these parameters between individuals according to age and sex. Height SDS, weight SDS, BMI SDS, and WC SDS, were determined through the LMS methods using the 2017 Korean reference^22^. SBP (mmHg) and DBP (mmHg) were measured three times on the right upper arm using a calibrated sphygmomanometer (Baumanometer Desk model 0320, Baum, NY, USA) and an appropriately sized cuff. BP was measured at 2-min intervals, and the mean of the last two BP measurements was used for analysis. Then, SBP and DBP were classified into the following four groups according to the Korea national reference: (1) < 50th percentile, (2) ≥ 50th and < 90th percentile, (3) ≥ 90 and < 95th percentile and (4) ≥ 95th percentile^22^.

Venous blood samples were collected after the participants fasted for at least 8 h. Blood samples were immediately processed, refrigerated, and transported to a central laboratory (NeoDin Medical Institute, Seoul, Korea) for analysis within 24 h. Routine biochemistry tests, including analyses of T-C, HDL-C, TGs, and glucose levels, were measured enzymatically using a Hitachi 7600 automatic analyzer (Hitachi, Tokyo, Japan). LDL-C was determined with Friedewald’s equation^29^.

### Collection of lifestyle-related parameter and socioeconomic status data

Information on lifestyle-related parameters and socioeconomic status was collected via questionnaires. Smoking, alcohol intake, and physical activity were included in this study as lifestyle-related parameters. Smokers were defined as individuals who smoked more than a total of five packs of cigarettes throughout their life, and subjects were divided into the following two groups: smokers and nonsmokers. Alcohol intake was defined as consuming at least two alcoholic beverages/month during the previous year, and subjects were divided into the following two groups: alcohol drinkers and nondrinkers. Physical activity was defined as meeting at least one of the following three criteria: (1) intense physical activity for 20 min at least three days/week, (2) moderate physical activity for 30 min at least five days/week, or (3) walking for 30 min at least five days/week. Subjects were then categorized into the following two groups based on physical activity: exercise or no exercise. Household income and residence were included as parameters of socioeconomic status. Household income was reported in quartiles, and subjects were categorized into the following two groups: the lowest quartile and the second quartile or higher. Residences were categorized according to location into the following two groups: urban and rural.

### Definitions of MetS and its components

Elevated WC was defined as a WC greater than or equal to the 90th percentile for sex and age according to 2017 Korean growth charts^22^. Elevated BP was defined as an SBP or DBP greater than or equal to the 90th percentile for sex, age, and height according to 2017 Korean growth charts^22^ or current administration of antihypertensive drugs. Elevated glucose was defined as fasting glucose concentrations ≥ 110 mg/dL or a previous diagnosis of type 2 diabetes mellitus (T2DM). T2DM was diagnosed in children and adolescents who met at least one of the following three criteria: (1) subjects who self-reported their disease using a questionnaire comprised of questions with yes or no answers, (2) children and adolescents currently using medications or receiving insulin to manage T2DM, or (3) participants with a fasting glucose level of at least 126 mg/dL during the national survey period. Elevated TGs were defined as serum TG concentrations ≥ 110 mg/dL or current administration of drugs for dyslipidemia, whereas reduced HDL-C was defined as serum HDL-C < 40 mg/dL. MetS was defined as the presence of at least three of the following five criteria: (1) elevated WC, (2) elevated BP, (3) elevated glucose, (4) elevated TGs, and (5) reduced HDL-C according to the modified criteria of the National Cholesterol Education Program Adult Treatment Panel III (NCEP ATP III)^30^.

### Definition of the WHtR groups according to age and sex

A previous study showed the 85th and 95th percentiles of WHtR as optimal cut-offs for predicting central obesity^11^. In addition, to assess the relationship between WHtR percentiles and cardiometabolic risk compared to the relationship with obesity diagnosis using BMI, which defined overweight as the ≥ 85th and < 95th percentiles and obesity as the ≥ 95th percentile in children and adolescents, subjects in this study were classified into three groups according to sex- and age-specific WHtR percentiles: (1) < 85th percentile, (2) ≥ 85th and < 95th percentile, and (3) ≥ 95th percentile.

### Statistical analysis

All analyses were conducted using R statistical package version 3.5.1 (The R Foundation for Statistical Computing, Vienna, Austria). Clinical characteristics are presented according to sex. Normally distributed continuous variables are presented as the means ± standard deviations (SDs), whereas categorical variables are presented as frequencies and percentages (%). Differences between boys and girls were analyzed using the independent *t*-test for normally distributed continuous variables and the chi-square (χ^2^) test for categorical variables. Sex- and age-specific reference data for WHtR were obtained by the LMS model to fit smoothed L (skew), M (median), and S (coefficient of variation) curves using gamlss package version 4.2.6. We presented the following percentile curves for WHtR in boys and girls: 3rd, 5th, 10th, 15th, 25th, 50th, 75th, 85th, 90th, 95th and 97th. The adjusted mean values of cardiometabolic risk factors were compared between the three groups (WHtR < 85th percentile, ≥ 85th and < 95th percentile, and ≥ 95th percentile) using analysis of covariance (ANCOVA) with Bonferroni’s post hoc test after adjusting for sex, age, BMI SDS, alcohol consumption, smoking, physical activity, residence, household income, and diagnosis of hypertension, T2DM, and dyslipidemia. The adjusted ORs and 95% confidence intervals (CIs) were estimated between WHtR groups, while the WHtR < 85th percentile group served as the reference group in multiple logistic regression analysis after adjusting for sex, age, BMI SDS, alcohol consumption, smoking, physical activity, residence, household income, diagnosis of hypertension, T2DM, and dyslipidemia. *P* < 0.05 was considered to indicate statistical significance.
